# Motor deficits and brain pathology in the Parkinson’s disease mouse model hA53Ttg

**DOI:** 10.3389/fnins.2024.1462041

**Published:** 2024-09-20

**Authors:** Livia Breznik, Magdalena Daurer, Roland Rabl, Tina Loeffler, Estibaliz Etxeberria-Rekalde, Joerg Neddens, Stefanie Flunkert, Manuela Prokesch

**Affiliations:** Scantox Neuro GmbH, Grambach, Austria

**Keywords:** Parkinson’s disease, α-synuclein, motor skills, neuroinflammation, neurodegeneration, transgenic mouse

## Abstract

**Background:**

Parkinson’s disease (PD) is a debilitating neurodegenerative disorder characterized by the progressive loss of dopaminergic neurons and the accumulation of α-synuclein (α-syn) aggregates. The A53T missense point mutation occurs in autosomal dominant familial PD and has been found to promote the aggregation of α-syn. To investigate the role of the A53T mutation in PD, researchers have developed various mouse models with this mutation.

**Objective:**

We therefore conducted a comprehensive characterization of the tg(THY1-SNCA*A53T)M53Sud mouse model (hA53Ttg mice) for its motor and pathological features.

**Methods:**

hA53Ttg mice were tested for motor impairments in a series of motor tests at 2, 4 or 6 months of age. Human α-syn and α-syn pSer129, as well as GFAP and Iba1 signal were labeled and quantified in the cortex, hippocampus, and brainstem. Neurofilament light chain (NF-L) levels were measured in the cerebrospinal fluid (CSF) and plasma. *Ex vivo* analyses were performed at the age of 2, 4, 6, and 10 months.

**Results:**

Behavioral tests revealed early muscle weakness and motor impairments that progressed with age. Immunohistochemical analyses demonstrated elevated levels of human α-syn and α-syn pSer129 in all evaluated brain regions. α-syn pSer129 labeling further revealed fiber-like structures in the cortex of older animals. Neuroinflammation was observed in an age-dependent manner. Biochemical evaluation revealed elevated NF-L levels in the plasma and CSF. Overall, our findings highlight the value of hA53Ttg mice in modeling PD-associated pathologies that closely resemble those observed in PD patients.

**Conclusion:**

Our results thus suggest that hA53Ttg mice are a useful tool for studying the underlying mechanisms of PD.

## Introduction

Parkinson’s disease (PD) is a chronic neurodegenerative disorder characterized by the progressive loss of dopaminergic neurons in the substantia nigra (SN) pars compacta and the presence of insoluble protein aggregates (Lewy bodies and Lewy neurites) primarily composed of α-synuclein (α-syn) (Colom-Cadena et al., 2017). PD is clinically diagnosed by progressive resting tremor, gait disturbance, postural instability, and bradykinesia/akinesia (Olanow and Tatton, 1999; Kouli et al., 2018). The exact pathogenesis of PD is not understood, however, an essential discovery for studies of pathogenesis highlights α-syn as an important element for familial and sporadic PD (Polymeropoulos et al., 1997; Spillantini et al., 1997; Calabresi et al., 2023). α-syn is an aggregation-prone, mainly presynaptic neuronal protein whose aggregates favorably bind to mitochondria and further decreases ATP production and induces fragmentation (Liu et al., 2009; Thorne and Tumbarello, 2022; Yi et al., 2022). Under pathological conditions, α-syn undergoes phosphorylation at serine 129 (Ser129). While this phenomenon typically occurs at minimal levels under physiological conditions, it significantly increases during pathological processes. In these instances, α-syn pSer129 is detected in approximately 90% of α-syn aggregates (Fujiwara et al., 2002; Muntane et al., 2012; Colom-Cadena et al., 2017). Ghanem et al. (2022) suggest that phosphorylation of Ser129 occurs after initial aggregation and possibly prevents the protein from further aggregation, thus having a protective function. Regardless of the exact function, the presence of α-syn pSer129 is widespread in the brain of patients suffering from synucleinopathies – a class of neurodegenerative diseases, such as PD and Lewy body dementia, displaying alterations of α-syn (Fujiwara et al., 2002; Parra-Rivas et al., 2023). α-syn seems to play a crucial role in the interaction between neuronal and immune system dysfunction in PD (Forloni, 2023). It has been shown that inflammation is a common feature in neurodegenerative diseases and a major driver of neurodegeneration (Garcia-Revilla et al., 2022; Kim et al., 2022; Basurco et al., 2023). Among brain cells, microglia and astrocytes are key players involved in inflammatory response. Studies in animal models have consistently reported that early microgliosis may precede the death of dopaminergic neurons, pointing towards an important role of the brain’s immune cells in the pathogenesis of PD (Garcia-Revilla et al., 2022). In recent years, neurofilament light chain (NF-L) has received great attention as a useful clinical biomarker for axonal damage in various neurodegenerative diseases. PD research has shown that NF-L levels correlate with disease progression and severity (Bäckström et al., 2020; Liu et al., 2022; Buhmann et al., 2023). The role of genetics in PD was long overlooked since most cases occur sporadically but about 5–10% of PD cases are familial inherited (Del Rey et al., 2018). The first mutation linked to PD was the alanine-53 to threonine (A53T) point mutation in the α-syn coding gene (SNCA). This mutation causes accelerated protein aggregation and neurotoxicity (Tambasco et al., 2016). Various α-syn transgenic mouse models were already generated utilizing different promoters. The murine Thy-1 promoter causes widespread transgene expression. While nigral dopaminergic cell loss is rarely observed, mice exhibit a range of motor symptoms and extensive α-syn pathology that commonly starts early (van der Putten et al., 2000; Maki et al., 2019). When comparing effects of wild type α-syn and A53T α-syn, it appears that the latter causes faster aggregation of α-syn and connected neurotoxicity (Magen and Chesselet, 2010; Kang et al., 2011). Thus, mouse models expressing human A53T mutation under control of the Thy1 promoter appear as a promising tool for studying disease progression and testing potential treatment strategies.

In this article, we thus present a comprehensive characterization of the tg(THY1-SNCA*A53T)M53Sud (hA53Ttg) mouse model, developed by T. C. Südhof and colleagues that was first described by Chandra et al. (2005). Previous research already showed that this model mimics key aspects of the disease (Gan et al., 2012; Rothman et al., 2013; Martin et al., 2014; Maki et al., 2019).

The aim of this study was to carry out a behavioral, histological, and biochemical characterization of hA53Ttg mice. Two, 4, and 6 months old hA53Ttg animals and their non-transgenic (ntg) littermates were therefore analyzed in a series of behavioral tests, including RotaRod, wire hanging, pasta gnawing, and beam walk. Quantitative immunofluorescent labeling was performed in the cortex, hippocampus, and brainstem for α-syn, α-syn pSer129, microgliosis and astrogliosis. Biochemical evaluation of NF-L levels in the CSF and plasma was conducted. In addition to aforementioned age groups, immunofluorescent experiments and evaluation of NF-L levels were performed in 10-month-old animals.

## Materials and methods

Eighty-eight hA53Ttg (JAX stock nr: 008135) mice and 87 non-transgenic (ntg) littermates bred at the AAALAC-accredited animal facility of Scantox Neuro (Grambach, Austria) were included in the study. Genotyping was performed before group allocation by collecting the ear punch during ear marking.

All animals were housed in single-sex groups of 3–5 mice per cage and kept in ventilated cages on standard rodent bedding (Rettenmaier^®^, Wilburgstetten, Germany) under a 12:12 h dark–light cycle and a temperature of 21 ± 1°C and 40 to 70% humidity. A cotton nestlet (Plexx, Elst, Netherlands) and wood wool (Rettenmaier^®^, Wilburgstetten, Germany) were added as enrichment material. Dried pelleted standard rodent chow (Altromin^®^, Lage, Germany) and water were provided *ad libitum*. All behavioral tests were performed during the light cycle phase (07:00 a.m. − 4:00 p.m.). Animals were allocated to experimental groups (50,50 male to female ratio, 23–24 animals per group) according to their genotype and initial body weight. All animals were randomly assigned to starting groups (cohorts) comprising animals of all groups. The number of animals in a cohort was limited to ensure uniform handling.

Animal’s body weight was recorded every 7 days throughout the study. Veterinarian care was provided throughout the study. One 6 months old male hA53Ttg mouse was prematurely euthanized due to paralysis and it was therefore excluded from the study.

Animal studies conformed to the Austrian guidelines for the care and use of laboratory animals (Tierversuchsgesetz 2012-TVG 2012, BGBl. I Nr. 114/2012). Animal housing and euthanasia were approved by the Styrian government (Amt der Steiermärkischen Landesregierung, Abteilung 13 – Umwelt und Raumordnung Austria; ABT13-78Jo323-2020).

### Experimental design

A cross-sectional study design with two factors (genotype and age) was employed. A total of 143 hA53Ttg and ntg littermates underwent a behavioral assessment at the age of 2, 4, or 6 months, followed by euthanasia for subsequent biochemical and histological analyses. Moreover, an additional group of 8 animals from each genotype, aged 10 months, was included solely for biochemical and histological evaluation.

### Wire hanging

The wire hanging test was performed to assess neuromuscular abnormalities of motor strength (Aartsma-Rus and van Putten, 2014; Hoffman and Winder, 2016). For the test, a wire cage lid was taped with duct tape around the perimeter to prevent the mouse from walking off the edge. The animal was placed on the cage grid which was subsequently slightly shaken so the animal tightly grabbed the wires. Then, the grid was turned upside down and placed on the holder approximately 50 cm above a soft underlay. This distance was high enough to prevent the mouse from jumping down, but not high enough to cause harm in the event of a fall. The latency to fall off the grid was measured in seconds. A 300 s cut-off time was used.

### Beam walk

The beam walk test was used to assess motor coordination and balance of mice by measuring the ability of the mice to traverse a graded series of narrow beams to reach the home cage (Carter et al., 2001). The wooden beams were 1 m long with a 20, 13, or 10 mm, square cross-section or a 28, 16, or 11 mm round diameter. The beams were placed horizontally, 50 cm above the surface, with one end mounted on a narrow support and the other end attached to the home cage into which the mouse could escape. An angle poise light was positioned above the start of the beam. Three training trials were performed prior to testing with three different starting points on the 20 mm square beam: close to the home cage (trial 1), in the center of the beam (trial 2), and at the brightly illuminated end of the beam (trial 3). Once the mice were trained, they received consecutive testing trials on each of the square beams and each of the round beams (except the 20 mm square training beam), in each case progressing from the widest to the narrowest beam. The testing trials were video recorded and afterwards evaluated with the Observer XT 10.5. software (Noldus, Wageningen, The Netherlands). The latency to traverse each beam and the number of times a hind foot slept off a beam were recorded for each trial.

### RotaRod

The RotaRod test was used to assess motor coordination in all mice (Dunham and Miya, 1957; Monville et al., 2006). Animals were placed on a rotating rod (four-lane-RotaRod; Ugo Basile, Gemonio, Italy) that ran at an accelerating speed starting at 2 revolutions per minute (rpm) reaching final speed of 20 rpm in 180 s. If a mouse lost its balance and fell onto an underlying platform, the rod automatically stopped and recorded the latency to fall. Prior to the testing, animals were trained to stay on the rod turning at a constant speed of 2 rpm for 1 min. Three trials were performed for each animal which were used to calculate the mean latency of each animal to stay on the rotating rod.

### Pasta gnawing

The test was performed to detect fine motor deficits (Rabl et al., 2016). Two days prior to testing, animals received a small piece of dry spaghetti daily to become familiar with the novel food. On the testing day, animals were brought to the experimental room 2 h prior to testing for habituation. The illumination during habituation and testing was 30–60 Lux. For testing, animals were individually placed on the testing table, the cage top, water bottle and food pellets were removed, and multiple pieces of spaghetti approximately 10 mm long were put in the center of the cage. A microphone (UltraSoundGate Condenser CM16; Avisoft Bioacoustics, Nordbahn, Germany) was positioned directly above the spaghetti piece. The recording was initiated as soon as the animal started to eat. Recording time was approx. 1 min. The number of bites per gnawing episode was evaluated using SASLab Pro (Avisoft Bioacoustics, Nordbahn, Germany).

### Tissue sampling and preparation

Animals were terminally anesthetized by intraperitoneal injection of 600 mg/kg pentobarbital. CSF and blood were collected. Then, animals were transcardially perfused with 0.9% physiological saline followed by brain dissection. The right hemispheres were subsequently fixed by immersion in freshly prepared 4% paraformaldehyde in phosphate buffer saline (PBS; pH = 7.4) for 2 h at room temperature and cryoprotected in 15% sucrose/PBS at 4°C. Afterwards, brain hemispheres were embedded in Tissue-Tek optimal cutting temperature (O.C.T.™, Leica Biosystems, item # 14020108926) compound in cryomolds, and then snap-frozen in dry ice-cooled liquid isopentane. Sagittal 10 μm thick cryosections from different medio-lateral brain levels – 0.54, 1.14, 1.74, 2.34, and 2.94 mm according to the brain atlas of Paxinos and Franklin (2001) – were generated on a Leica cryotome.

### Immunofluorescent labeling

Five cryosections per animal, 1 from each medio-lateral brain level, were pre-treated with freshly prepared cooled sodium borohydride (1 mg/mL, Sigma-Aldrich, #213462) for 4 min and then incubated with proteinase K (12.5% in tris buffered saline, Dako, #S3020) for 20 min at 37°C. Afterwards, tissue sections were subsequently treated with 10% donkey serum/0.1% Triton X-100/PBS for 1 h and incubated with primary antibodies listed in Table 1 for 2 days at 4°C. Primary antibodies were visualized by incubating sections with secondary antibodies shown in Table 2 for 1 h at room temperature. Cell nuclei were labeled with DAPI working solution (item # A1001, Pan Reac AppliChem GmbH, Darmstadt, Germany). Finally, sections were covered with Mowiol^®^ 4–88 (#81381, Sigma-Aldrich, St. Louis, MO, United States) and coverslips. Throughout the experiment, tissue sections were washed with PBS for 5 min at least twice between individual steps. All steps during and after the use of fluorophore-conjugated antibodies were performed in the dark.

**Table 1:** List of primary antibodies used for immunofluorescent labeling.

Antigen	Clone	Vendor	Item #	Dilution*
a-synuclein (human)	15G7	Enzo Life Sciences	ALX-804-258	1/10
Alpha-synuclein (phospho S129)	EP1536Y	Abcam	ab51253	1/1000
GFAP	Polyclonal	Abcam	ab53554	1/1000
IBA1	Polyclonal	Synaptic systems	234,004	1/2000

*Diluted in 1% donkey serum/PBS.

**Table 2:** List of secondary antibodies used for immunofluorescent labeling.

Secondary antibodies	Vendor	Item #	Dilution*
Donkey Anti-Rat IgG H&L (Alexa Fluor^®^ 647) preadsorbed	Abcam	ab150155	1/500
Donkey Anti-Rabbit IgG H&L (Alexa Fluor^®^ 750) preadsorbed	Abcam	ab175728	
Donkey Anti-Sheep IgG H&L (Alexa Fluor^®^ 488)	Abcam	ab150177	
Cy™3 AffiniPure™ Donkey Anti-Guinea Pig IgG (H + L)	Jackson ImmunoResearch Laboratories	706-165-148	

*Diluted in 1% donkey serum/PBS.

### Imaging and quantitative image analysis

Mosaic images of the immunofluorescently labelled sections were recorded on a Zeiss automatic microscope AxioScan Z1 with high aperture lenses, equipped with a Zeiss Axiocam 506 mono and a Hitachi 3CCD HV-F202SCL camera and Zeiss ZEN 2.3 software.

Quantitative image analysis was performed with Image-Pro 10 (Media Cybernetics). The regions of interest (ROIs) – cortex, hippocampus, and brainstem – were individually identified on each section.

After defining intensity threshold, morphological size, and shape filter parameters for detection of the targeted signal, quantification of immunolabeling was performed automatically using a macro and thus the same parameters on all images. This rater-independent approach enabled determining the percentage of ROI area covered by immunoreactive (IR) signal, so that the resulting values are normalized for ROI size. Also, different object features were then quantified, among them the number of objects normalized to ROI size (mean object density), the mean signal intensity of identified objects (mean object intensity) and the size of above-threshold objects (mean object size).

### Neurofilament light chain levels in plasma and cerebrospinal fluid

For the measurement of NF-L levels in terminal plasma and CSF samples, the NF-light R^©^ ELISA 10–7,001 CE (UmanDiagnostics, Umeå, Sweden) was used. Specificity of the assay for murine samples was already shown by Bacioglu et al. (2016) using NF-L deficient mouse samples as control. Samples were diluted 1:3 and 1:30 in assay buffer for plasma and CSF samples, respectively. The assay was performed in duplicates and according to the manufacturer’s protocol on the Cytation 5 multimode reader (Biotek, Winooski, VT, United States). Data were evaluated in comparison to calibration curves provided by the manufacturer and are expressed as pg/mL plasma. All analyzed samples from hA53Ttg mice were well within the detection range of the assay, only some samples from ntg animals were close to or below the lowest limit of quantification (LLOQ).

### Statistical analyses

Statistical analyses and graph preparations were conducted using GraphPad Prism (version 10.1.2, San Diego, CA, United States). Descriptive statistical analyses were performed for all parameters. Group variances were calculated using two-way ANOVA. If a significant effect was detected, Bonferroni’s *post hoc* analysis was employed. In the beam walk test, the number of slips and active time on the fifth beam were analyzed using the Mann–Whitney *U*-test. Fisher’s exact test was utilized to compare the number of performers in the beam walk test. Pearson’s correlation coefficient (*r*) was used to examine the relationship between plasma NF-L levels and α-syn pSer129 immunoreactive area in the brainstem.

Raw data of all experiments are provided in Supplementary Table S1.

A detailed description of performed statistical analyses is given in the corresponding figure legend. Data were averaged and are presented as mean ± standard error mean (SEM). An α-error level of *p* < 0.05 was considered significant.

## Results

Behavioral outcomes of males and females were initially analyzed separately. Only minor differences were found, and results were therefore merged and reported as mixed sex.

### Progressive motor impairments in hA53Ttg mice

For assessing motor impairments in hA53Ttg animals, a series of behavioral tests was conducted, namely the wire hanging, beam walk, pasta gnawing, and RotaRod test.

**Figure 1:**
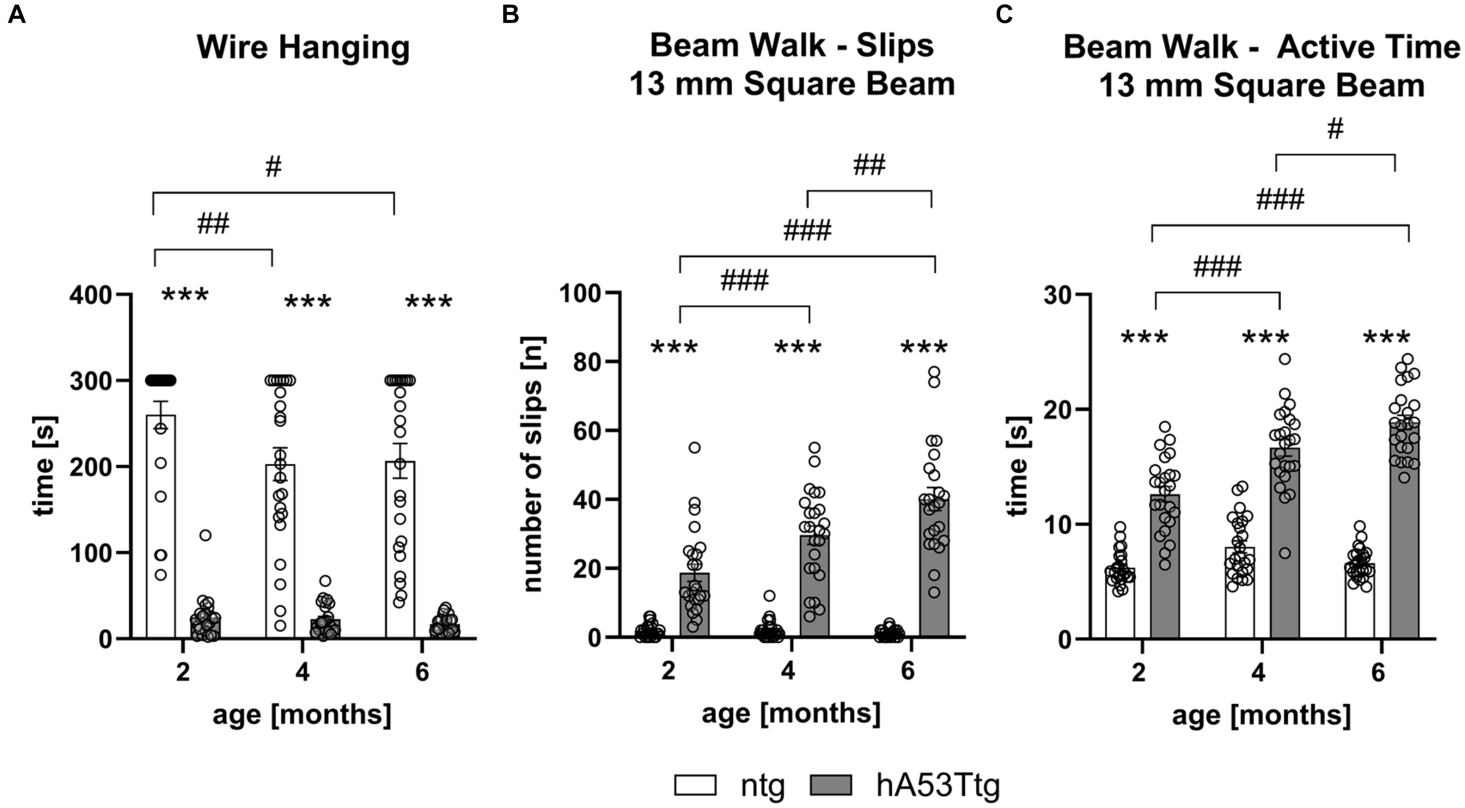
Decreased muscle strength and motor coordination in hA53Ttg mice. The wire hanging test **(A)** shows a reduced latency to fall while the beam walk test shows an increased number of slips **(B)** and an increased time **(C)** needed to traverse the 13 mm square beam in hA53Ttg animals in comparison to ntg controls; *n* = 23–24 per group. Mean ± SEM; Two-way ANOVA followed by Bonferroni’s *post hoc* test; #*p* < 0.05, ##*p* < 0.01, ***/###*p* < 0.001. *Differences between genotypes; #differences between age groups.

The wire hanging test revealed that hA53Ttg animals exhibited a severely reduced latency to fall from the wire at all three inspected ages: 2, 4, and 6 months of age (*p* < 0.001 for all comparisons) compared to age-matched ntg littermates. There was an additional significant decrease in the hanging time of 4- and 6-month-old ntg animals compared to 2-month-old ntg animals (*p* = 0.009 and 0.02, respectively; Figure 1A).

The beam walk test revealed an impaired performance already at 2 months of age (Figures 1B,C and Supplementary Figures S1, S2). Results indicate that hA53Ttg animals performed more slips (Figure 1B and Supplementary Figure S1) on the first 4 beams (square 13- and 10-mm beams and round 28- and 16-mm beams) at all three evaluated ages (*p* < 0.001 for all comparisons, except for the round beam 28 mm hA53Ttg compared to 2 months old ntg mice; *p* = 0.005). Moreover, 4- and 6-month-old hA53Ttg animals showed an increased number of slips over time on the first four beams compared to 2-month-old hA53Ttg mice (*p* < 0.001), and when comparing 4-month-old to 6-month-old hA53Ttg animals (*p* = 0.01, 0.03 for 13- and 10-mm square beams and *p* < 0.001 for the 28- and 16-mm round beams; Figures 1B,C and Supplementary Figures S1, S2). In addition to the increased number of slips, hA53Ttg mice also required more time (Figure 1C and Supplementary Figure S2) to cross the first 4 beams at all three evaluated ages (*p* < 0.001).

An age-dependent increased time to cross the beam was observed in hA53Ttg animals on the first 4 beams (Table 3). In contrast, no such increase was observed in ntg animals.

**Table 3:** Age-dependent increase of active time in the beam walk test.

Compared groups	Beam			
	13 mm square	10 mm square	28 mm round	16 mm round
2 vs. 4 months	*p* < 0.001	*p* < 0.001	*p* < 0.001	*p* < 0.001
2 vs. 6 months	*p* < 0.001	*p* < 0.001	*p* < 0.001	*p* < 0.001
4 vs. 6 months	*p* = 0.01	*p* = 0.003	*p* < 0.001	*p* < 0.001

*p-values comparing age groups of hA53Ttg mice on different beams.*

**Figure 2:**
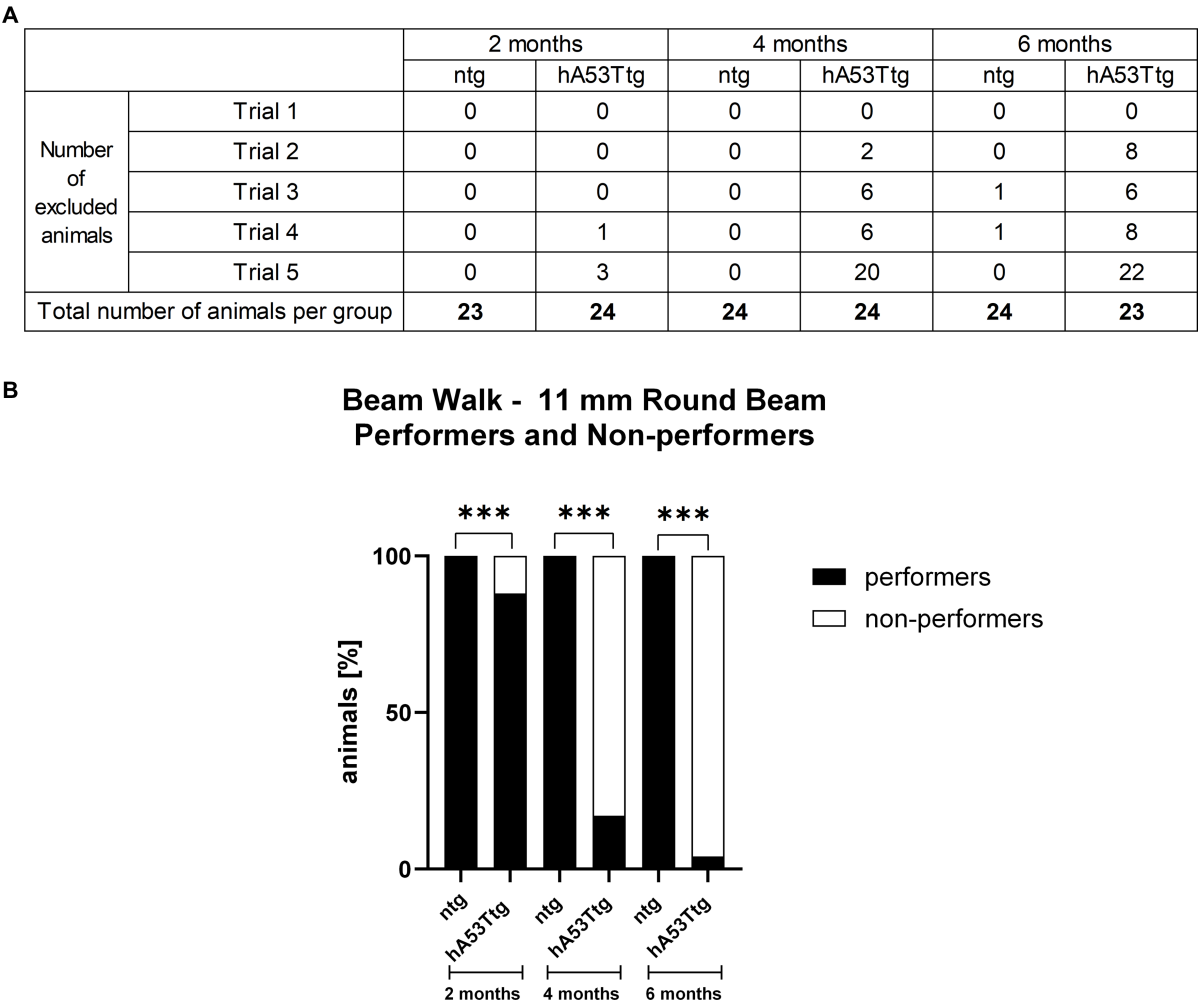
Performers and non-performers in the beam walk test. Number of animals excluded from the beam walk test evaluation due to a fall from the beam **(A)**. Rows display the number of excluded animals for each trial while columns display the number of excluded animals per group. The bottom row contains the total number of animals tested per group. Analysis of performance showed that less hA53Ttg animals were able to traverse the beam at any age compared to ntg mice **(B)**. Number of animals in percent; *n* = 23–24 per group. Fisher’s exact test; ***p < 0.001.

When assessing the performance on the most challenging beam (11 mm round beam; Figure 2B and Supplementary Figures S1D, S2D), it was only possible to compare the number of slips of hA53Ttg mice at the age of 2 months, as the majority of 4- and 6-month-old hA53Ttg mice fell off the beam (Figure 2A). At 2 months of age, hA53Ttg animals exhibited an increased number of slips (*p* < 0.001) on the 11 mm round beam (Supplementary Figure S1D). Animals that fell off the beam before completely traversing the beam were excluded from the analysis and called non-performers (Figure 2A). The number of performers and non-performers was examined (Figure 2B) showing that already at the age of 2 months, significantly more hA53Ttg mice were non-performers compared to ntg mice and thus not able to completely cross the beam (*p* < 0.001). This difference persisted over age (*p* < 0.001 for both comparisons).

**Figure 3:**
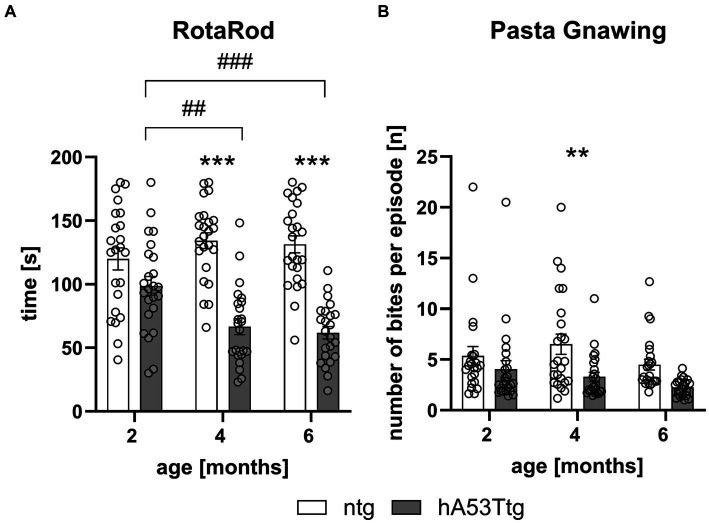
Motor coordination and orofacial muscle impairment in hA53Ttg mice. Analysis of animals showed a reduced latency to fall in the RotaRod test **(A)** and a reduced number of bites per episode in the pasta gnawing test **(B)** starting at 4 months of age in hA53Ttg animals compared to ntg mice; *n* = 23–24 per group. Mean ± SEM; two-way ANOVA followed by Bonferroni’s *post hoc* test; **/##p < 0.01, ***/###p < 0.001. *Differences between genotypes; #differences between age groups.

Evaluation of hA53Ttg mice in the RotaRod test showed a reduced mean latency to stay on the rotating rod at 4 (*p* < 0.001) and 6 months of age (*p* < 0.001) compared to age-matched ntg animals. A significant effect of the time animals needed to traverse the beam was observed for hA53Ttg animals when comparing 2- vs. 4- and 6-months old animals (*p* = 0.003 and *p* < 0.001, respectively; Figure 3A).

The pasta gnawing test revealed a decrease in the number of bites per episode at 4 months of age in hA53Ttg mice compared to age-matched ntg mice (*p* = 0.01) (Figure 3B).

### Human and phosphorylated α-synuclein levels in hA53Ttg mice

**Figure 4:**
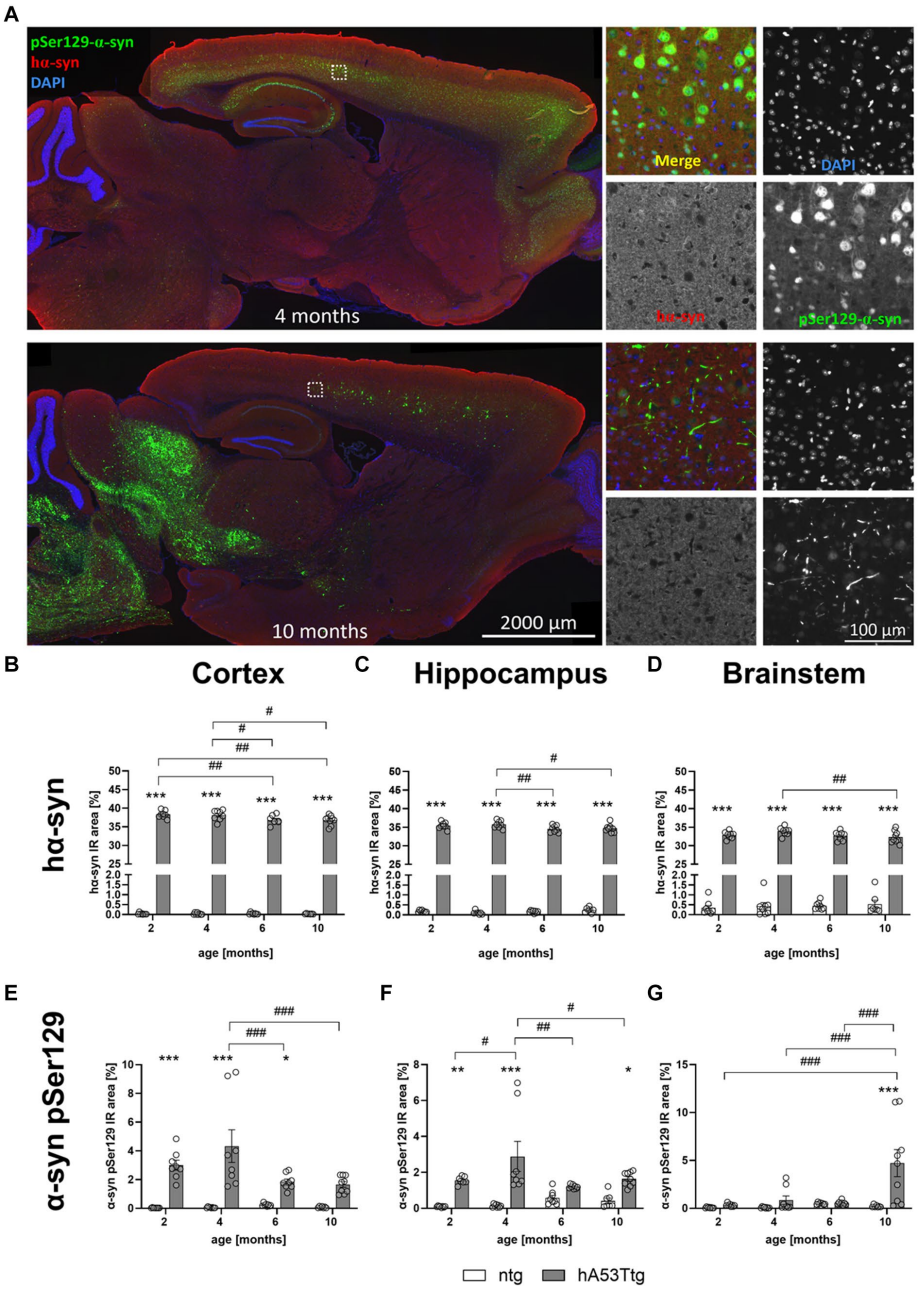
Human and phosphorylated α-synuclein in different brain regions. **(A)** Labeling of α-syn pSer129 (green) and hα-syn (red) in the cortex of hA53Ttg mice at the age of 4 and 10 months; cell nuclei were labeled with DAPI (blue). Mice at 4 months display α-syn pSer129 labeling which is restricted to nuclear and somatic location in the cortex, whereas the nuclear/somatic pSer129 α-syn labeling becomes weaker in 10 months old mice. However, note the appearance of high intensity signal which resembles a more fiber-like pattern at this age. Single channel images were taken at the position indicated by the rectangle in the left overview image. Increased immunoreactive (IR) area of human α-synuclein (hα-syn) in the cortex **(B)**, hippocampus **(C)** and brainstem **(D)** in hA53Ttg animals already at 2 months of age compared to ntg littermates. IR area of α-synuclein phosphorylated at serine 129 (α-syn pSer129) in the cortex **(E)**, hippocampus **(F)** and brainstem **(G)**; *n* = 7–9 per group. Mean ± SEM; two-way ANOVA followed by Bonferroni’s *post hoc* test; */#p < 0.05, **/##*p* > 0.01, ***/###p < 0.001. *Differences between genotypes; #differences between age groups.

Representative images of human α-syn and α-syn pSer129 labeling are shown in Figure 4A. At the age of 4 months, α-syn pSer129 labeling is restricted to nuclear and somatic locations in the cortex. Although nuclear/somatic α-syn pSer129 labeling becomes weaker in 10 months old mice, the signal is of higher intensity and thus resembling a more fiber-like pattern (see higher magnification images in Figure 4A).

To evaluate levels of human α-syn (hα-syn), the immunoreactive (IR) area within the cortex (Figure 4B), hippocampus (Figure 4C), and brainstem (Figure 4D) of hA53Ttg and ntg mice were evaluated at the age of 2, 4, 6, and 10 months. The analysis revealed consistently elevated levels of human-specific α-syn in all analyzed brain regions of hA53Ttg mice (*p* < 0.001 for all comparisons). Within the hA53Ttg mice, a progressive decline in hα-syn levels was observed in all 3 brain regions. In the cortex, 6- and 10-month-old hA53Ttg animals presented lower α-syn levels than 2- (*p* = 0.06 and 0.04, respectively) and 4- (*p* = 0.03 and 0.02, respectively) month-old hA53Ttg animals. In the hippocampus, the α-syn IR area of hA53Ttg was decreased in 6- and 10-month-old animals compared to 4-month-old hA53Ttg mice (*p* = 0.009 and 0.02, respectively), while analysis within the brainstem revealed a decline in 10-month-old hA53Ttg mice compared to 4-month-old hA53Ttg mice (*p* = 0.008).

The quantitative analysis of α-syn pSer129 levels showed a notable increase in the IR area within the cortex (Figure 4E) of hA53Ttg mice at 2 (*p* < 0.001), 4 (*p* < 0.001), and 6 (*p* = 0.04) months of age compared to ntg littermates. A genotype effect was also evident in the hippocampus (Figure 4F), with an increase at the age of 2 (*p* = 0.009), 4 (*p* < 0.001), and 10 (*p* = 0.03) months, although not at 6 months. Furthermore, in the brainstem of hA53Ttg mice (Figure 4G), a marked increase in the α-syn pSer129 IR area was evident solely at 10 months of age compared to ntg littermates (*p* < 0.001). Within the hA53Ttg mice, an increased α-syn pSer129 IR area in the cortex was observed at 4 months of age compared to 6 and 10 months (both *p* < 0.001). Similarly, an increase in α-syn pSer129 IR area was evident in the hippocampus of 4-month-old hA53Ttg animals compared to mice at the age of 2 (*p* = 0.03), 6 (*p* = 0.002), and 10 (*p* = 0.03) months. Evaluation of the brainstem of hA53Ttg animals showed an overall increase in the α-syn pSer129 IR area at the age of 10 months compared to 2, 4, and 6 months (*p* < 0.001 for all comparisons; Figure 4G).

**Figure 5:**
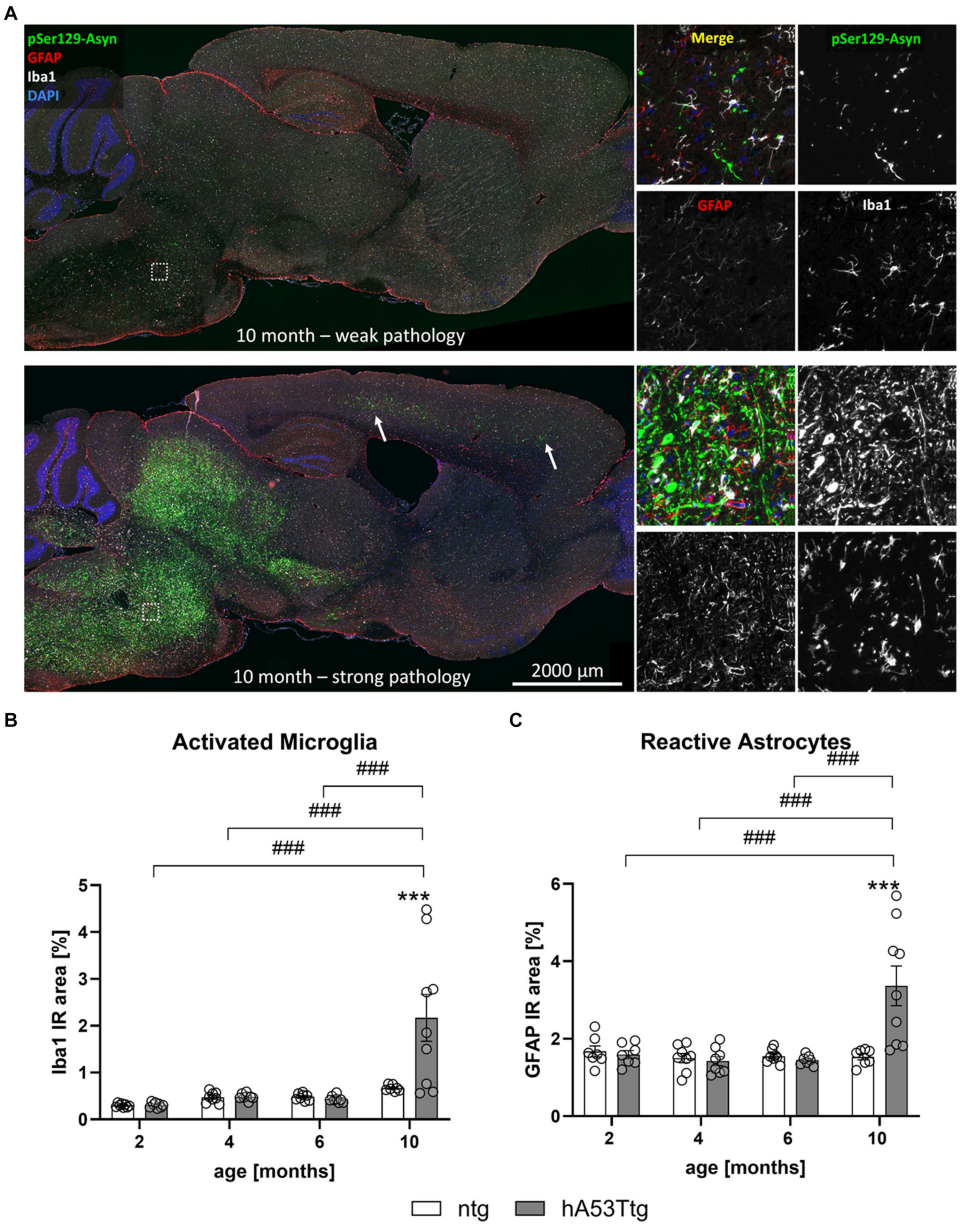
Neuroinflammation in the brainstem. **(A)** Labeling of pSer129 α-syn (green), GFAP (red), and Iba1 (white) shows striking differences between individual animals of the same age and genotype; cell nuclei were labelled with DAPI (blue). “Weak pathology” mice display little labeling for all three markers, whereas a large amount of pSer129 α-syn and associated gliosis is evident in the brainstem of “strong pathology” mice. Images thus support a high within-group variation of hA53Ttg mice at the age of 10 months. Single channel images were taken at the position indicated by the rectangle in the left overview image. Arrows point towards areas with strong pathology in the cortex. Increased immunoreactive (IR) area of ionized calcium binding adaptor molecule 1 (Iba1; **B**) and glial fibrillary acidic protein (GFAP; **C**) in the brainstem of 10-months-old hA53Ttg animals compared to ntg littermates; *n* = 7–9 per group. Mean ± SEM; two-way ANOVA followed by Bonferroni’s *post hoc* test; ***/###p < 0.001. *Differences between genotypes; #differences between age groups.

A detailed analysis of hA53Ttg mice showed a high variation of α-syn pSer219 levels in the brainstem of 10 months old animals – some displaying a weak pathology while others displaying a strong pathology (Figure 5A). Although variations in pathology were quite high, no animals were excluded from the analysis.

### Inflammation in different brain regions of hA53Ttg mice

Immunofluorescent labeling of ionized calcium binding adaptor molecule 1 (Iba1) and glial fibrillary acidic protein (GFAP) as a marker for microgliosis and astrogliosis, respectively, was performed in the brain of 2-, 4-, 6-, and 10-month-old hA53Ttg mice and corresponding ntg littermates.

Representative images of GFAP, Iba1 and α-syn pSer129 labeling in the brains of 10-month-old hA53Ttg mice are shown in Figure 5A. A notable interindividual variability in the pathological expression is observed within mice of the same age and genotype. The upper panel displays an example of weak pathology, characterized by lower levels of α-syn pSer129, while the lower panel presents an example of strong pathology, defined by markedly higher levels of α-syn pSer129. Quantification of the immunofluorescent Iba1 signal in the brainstem (Figure 5B) showed a significant increase of the IR area in 10-month-old hA53Ttg mice compared to age-matched ntg littermates (*p* < 0.001). In detail, object size, intensity, and density of Iba1-positive objects (Supplementary Figure S3) was increased in 10-month-old hA53Ttg animals compared to ntg littermates (*p* < 0.001, *p* = 0.01, *p* < 0.001, respectively). Comparisons across age groups of hA53Ttg mice resulted in an increased IR area (Figure 5C), as well as object size, intensity, and density (Supplementary Figure S3) in 10-month-old hA53Ttg mice compared to mice at the age of 2, 4, and 6 months (*p* < 0.001 for all comparisons; except object intensity of 4- vs. 10-month-old hA53Ttg animals was *p* = 0.001). Overall, a severe progression of microgliosis was observed between the age of 6 and 10 months in the brainstem of hA53Ttg mice combined with a high variation (Figure 5). Evaluation of Iba1 IR area in the cortex (Supplementary Figures S4A,B) and hippocampus (Supplementary Figures S4C,D) of hA53Ttg mice showed no significant differences compared to ntg littermates. However, a significantly higher Iba1 IR area was observed within the cortex of 10-month-old hA53Ttg animals compared to 2- (*p* < 0.001) and 6- (*p* = 0.001) month-old genotype-matched animals. A similar progressive increase was observed in ntg littermates, with 10-month-old animals differing from 2-month-old ntg mice (*p* < 0.001). In the hippocampus, an elevated Iba1 IR area was observed in 4-months-old hA53Ttg animals compared to 2- (*p* = 0.02) and 6- (*p* = 0.03) month-old animals of the same genotype. When analyzing the hippocampus of ntg mice, a similar trend was observed as in the cortex – a significant increase in the Iba1 IR area of 10-month-old animals compared to 2-month-old ntg animals (*p* < 0.001).

Intriguingly, GFAP IR area (Figure 5C) was significantly increased in the brainstem of 10-month-old hA53Ttg mice compared to ntg mice (*p* < 0.001) as well as of all younger genotype-matched animals (*p* < 0.001). A more detailed analysis showed that the object density of GFAP-positive objects (Supplementary Figure S5C) – number of astrocytes – was elevated in comparison with ntg littermates and all younger hA53Ttg animals (*p* < 0.001). Similar as for Iba1, a severe progression of astrogliosis was observed between the age of 6 and 10 months in the brainstem of hA53Ttg mice, although displaying a high variation in pathology within the group (Figure 5A). We attempted to determine the potential cause for this observation and investigated whether there was any correlation between α-syn pSer129 burden and the litter or sex of the individuals. However, we were unable to identify any variable that could explain this finding. In the hippocampus, no differences between genotypes were observed at any given time. However, at the age of 2 months, hA53Ttg mice exhibited a higher GFAP IR area (Supplementary Figure S6C) compared to 4- (*p* = 0.004), 6- (*p* < 0.001), and 10- (*p* < 0.001) month-old hA53Ttg animals. The GFAP-positive object density (Supplementary Figure S6D) of 2-month-old hA53Ttg mice was increased compared to 6-month-old animals of the same genotype (*p* = 0.005). Cortical GFAP-positive IR area and object density were increased at 4 months of age (Supplementary Figures S6A,B) in hA53Ttg mice compared to ntg littermates (*p* = 0.004 and *p* = 0.002, respectively). Within the hA53Ttg group, a significant increase of the cortical GFAP IR area and object density was observed in 4-month-old animals in comparison to 6-month-old genotype-matched controls (*p* = 0.004 and *p* = 0.002, respectively).

### NF-L correlates with α-syn pS129 levels

**Figure 6:**
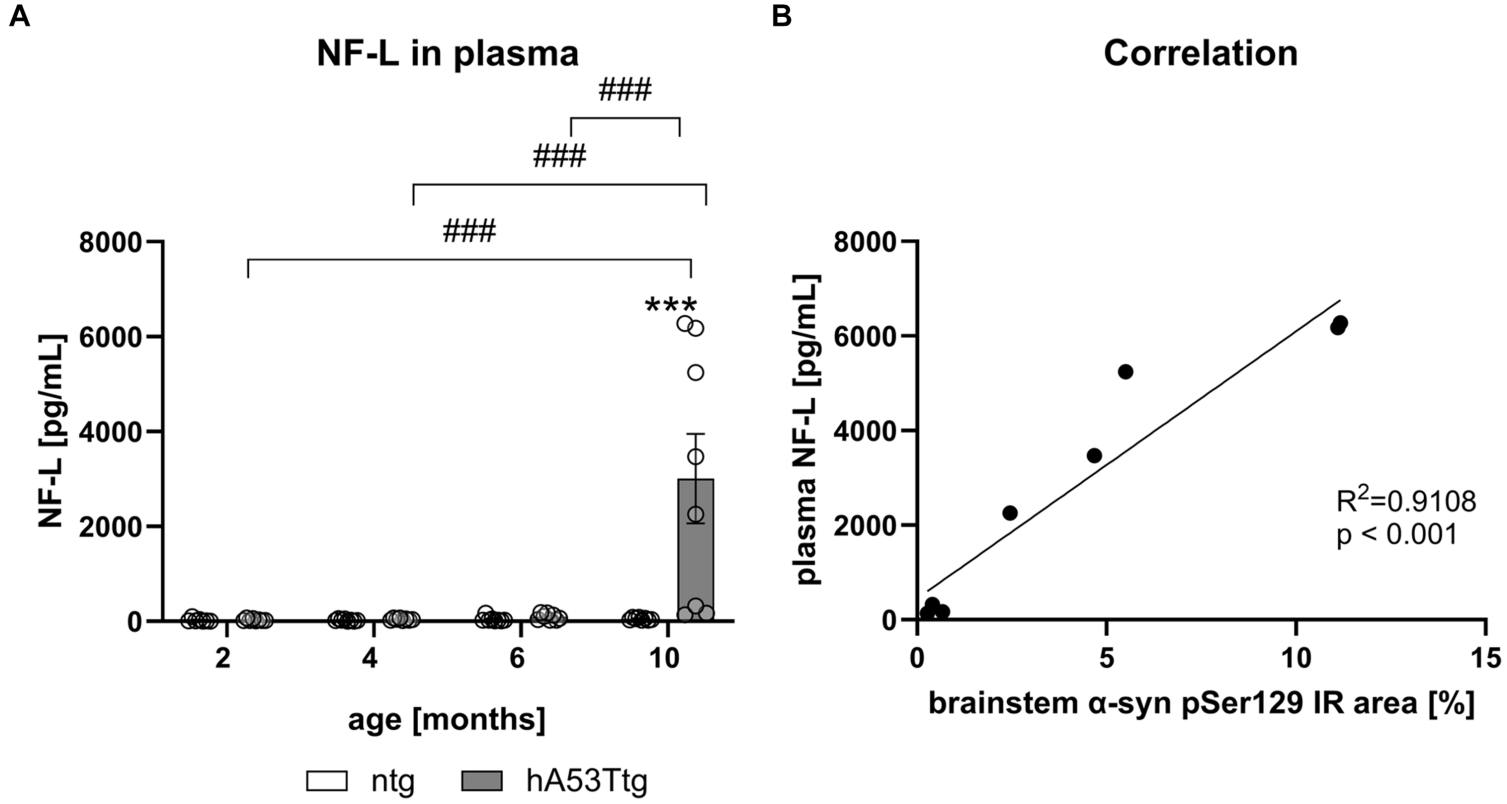
Quantification of NF-L levels in the plasma of hA53Ttg mice and correlation with α-syn pSer129. **(A)** Neurofilament-light chain (NF-L) levels in pg/mL in the plasma of hA53Ttg and ntg littermates at 2–10 months of age; *n* = 8 per group. Mean ± SEM; two-way ANOVA followed by Bonferroni’s *post hoc* test; ***/###*p* < 0.001. **(B)** High correlation between plasma NF-L levels and immunoreactive (IR) area of α-syn pSer129 in the brainstem of 10-months-old hA53Ttg mice. *Differences between genotypes; #differences between age groups.

Evaluation of NF-L levels was conducted in plasma (Figure 6A) and CSF (Supplementary Figure S7) samples of hA53Ttg mice and ntg littermates. No significant difference was observed between 2 to 6 months old hA53Ttg mice and ntg controls. However, a notable increase in NF-L levels emerged (*p* < 0.001) in 10-month-old hA53Ttg mice compared to age-matched ntg littermates. Furthermore, 10-month-old hA53Ttg animals exhibited increased NF-L levels compared to 2-, 4-, and 6-months-old hA53Ttg mice (*p* < 0.001 for all comparisons). A robust correlation (*R*^2^ = 0.9108, *p* < 0.001) was observed between NF-L levels in the plasma and α-syn pS129 IR area in the brainstem (Figure 6B). Due to this high correlation, animals with strong and weak pathology could be recognized not only by α-syn pS129 IR area, but also by NF-L levels in plasma.

## Discussion

Due to its complex etiology and debilitating symptoms, PD poses a significant challenge in the realm of neuroscience research. In this study, we conducted a comprehensive characterization of the hA53Ttg mouse model focusing on behavioral, histological, and biochemical aspects of PD pathology. To our knowledge, this is the first study presenting an in-depth characterization of the hA53Ttg mouse model over time.

### Progressive motor impairment

Our results show that hA53Ttg mice present decreased muscular strength and motor coordination already at the age of 2 months. By assessing motor coordination with the beam walk test and the RotaRod test, we observed that the beam walk test is more sensitive in detecting early motor coordination impairments as hA53Ttg mice already presented highly significant deficits at the age of only 2 months in this test. This is in line with previous reports, that show a higher sensitivity of the beam walk test compared to the RotaRod by evaluating various transgenic and induced rodent models in both tests (Stanley et al., 2005; Luong et al., 2011). Early impairment of motor coordination and muscle strength in the hA53Ttg mice was previously shown using the RotaRod, wire hanging test, and the beam walk test in 1.5–2 months old mice (Maki et al., 2019). Rothman et al. (2013) noted an impaired gait at 2 months of age while an impaired performance on the RotaRod was observed starting at the age of 6 months. Differences in the phenotype onset might be contributed to differences in testing protocols since Maki et al. (2019) trained animals on the RotaRod for 3 days before testing, while Rothman et al. (2013) report no such training period. Data by Rothman et al. (2013) are thus better comparable to our test protocol. Taken together, hA53Ttg mice exhibit early motor impairments that are first observed as muscle weakness and impaired motor coordination. The latter can be robustly detected with more sensitive tests, such as the beam walk test, while changes in the RotaRod performance are frequently observed at later age, when motor impairments worsen. Additionally, we have shown that hA53Ttg mice present orofacial impairments, which are of high translational value as reduced jaw mobility and impaired orofacial function is reported in PD patients (Friedlander et al., 2009; Puyjarinet et al., 2019; Baram et al., 2023). To our knowledge, we are the first to use the stress-free pasta gnawing test in this mouse model (Rabl et al., 2016).

When comparing results of hA53Ttg mice with mice that overexpress wild type α-syn under the same promoter, it seems that wild type α-syn mice present an even earlier motor phenotype. Rabl et al. (2017) reported a significantly reduced motor strength of Line 61 mice starting as early as 1 month of age, impairments in motor coordination measured with the RotaRod at 2 months of age, and impairments in the pasta gnawing test starting at 3 months of age. We therefore assume that the A53T α-syn mutation might slightly delay the onset of muscle weakness and motor coordination.

### α-synuclein

α-syn is a key element found in pathological occlusions of patients with synucleinopathies (Fujiwara et al., 2002; Muntane et al., 2012; Colom-Cadena et al., 2017). Increased levels of α-syn pSer129 in the CSF and various brain areas, including brainstem, limbic cortex and neocortex are observed in PD patients compared to healthy individuals (Rezaie et al., 1996; Majbour et al., 2016). We have shown a robust and consistent expression of human α-syn in the brainstem, cortex and hippocampus of hA53Ttg animals which aligns with already published results (Chandra et al., 2005; Martin et al., 2014). In addition, we evaluated α-syn pSer129 levels in various brain regions of hA53Ttg animals and observed fiber-like structures in the cortex. The humanized A53T mutation was previously shown to increase phosphorylation of α-syn on residue Ser129 in the brain of transgenic mice (Bencsik et al., 2014; Ma et al., 2023). We could not identify any study that already investigated α-syn pSer129 levels in the brain of hA53Ttg mice. However, Martin et al. (2014) used Syn211 monoclonal antibody to specifically label hα-syn with higher molecular weight in the brain of hA53Ttg animals and named it aggregates. Since it was previously shown that α-syn pSer129 is preferentially found in aggregates in the brain of PD patients and mice overexpressing α-syn, our observation of fiber-like structures in the cortex might indicate the existence of aggregates in hA53Ttg mice (Fujiwara et al., 2002; Neumann et al., 2002; Ghanem et al., 2022). Although further research is needed to validate this hypothesis. In summary, besides expressing high levels of mutated α-syn, the widespread presence of α-syn pSer129 in hA53Ttg mice is comparable to α-syn pSer129 expression in PD patients (Majbour et al., 2016).

### Inflammation

Inflammation, marked by the activation of astrocytes and microglia, plays a pivotal role in PD pathogenesis and is a commonly reported pathology in PD patients (Chen et al., 2023). Recent evidence suggests that in PD, early pathological processes are likely to be governed by inflammation activated by aggregated α-syn forms (Wang et al., 2023). In line with this, our results show the most striking phosphorylation of α-syn in the brainstem, which is also the most prominent brain area presenting astro-and microgliosis. We further examined astrogliosis and microgliosis in the striatum but did not find any differences between hA53Ttg mice and age-matched control animals (data not shown). Although we could not find any previous reports investigating inflammatory markers in the striatum of hA53Ttg mice, Gao et al. (2011) treated mice expressing the human A53T mutation driven by the mouse prion protein promoter with the inflammogen lipopolysaccharide (LPS). Their results show that mutant animals are more susceptible to LPS-induced neurotoxicity in the striatum and substantia nigra, suggesting that the A53T mutation makes animals more vulnerable to neurotoxins. Gan et al. (2012) demonstrated elevated levels of GFAP and Iba1 in the spinal cord of 6-month-old hA53Ttg animals that displayed PD-like symptoms. While our investigation did not extend to the spinal cord, we observed increased neuroinflammation in the brainstem of 10-month-old-animals. The discrepancy in the timing of inflammation onset compared to the study by Gan et al. (2012) could be explained by the fact that they evaluated inflammation only in animals that displayed PD-like symptoms, while we evaluated all animals. Alternatively, inflammation of the spinal cord could precede inflammation in the brain. However, we did observe a transient increase in astrogliosis in the cortex of younger animals. To determine the exact reason for these deviating findings, further research is needed. Interestingly, while we observed an increase in the number of GFAP-expressing astrocytes, microglia not only increased in number but also displayed a more pronounced activation, suggesting a potentiated inflammatory state which is in line with a previously reported central role of microglia in PD pathogenesis (Garcia-Revilla et al., 2022). On that note, Krzisch et al. transplanted A53T-mutant human iPSC-derived microglia into mouse brains and showed that the A53T mutation enhances proinflammatory activation (unpublished data). Our findings thus show that hA53Ttg mice exhibit locally increased micro-and astro-gliosis, and thus recapitulate some of the neuroinflammatory features observed in PD patients (Chen et al., 2023).

### Axonal degeneration

Although the loss of dopaminergic neurons in the SN is the most reported cause of motor impairments in PD, some authors challenged this view and suggested that it is in fact axonal degeneration that drives deterioration of the motor system and precedes neuronal loss (Tofaris et al., 2006; Liu et al., 2009; Burke and O'Malley, 2013; Kurowska et al., 2016; Gcwensa et al., 2021). NF-L is a highly sensitive and reliable biomarker for axonal damage, and it can predict motor and cognitive function in PD patients (Buhmann et al., 2023). We observed increased levels of NF-L in the plasma and CSF of aged hA53Ttg animals, with a high correlation between plasma NF-L levels and brainstem IR area of α-syn pSer129. The high correlation could indicate that axonal degeneration in hA53Tg mice originates in the brainstem, which was previously observed in PD patients (Chandra et al., 2005; Rothman et al., 2013; Martin et al., 2014; Oliveira et al., 2017; Pelz et al., 2018). Axonal degeneration in the hA53Ttg mice was reported also by others (van der Putten et al., 2000; Martin et al., 2014). van der Putten et al. (2000) used Holmes-Luxol staining to visualize the breakdown and segmentation of axonal myelin in the spinal roots of 6.5 months old animals, while Martin et al. (2014) showed cortical axonal degeneration in 12-months old hA53Ttg animals using silver staining. Since different methods were used, it is not possible to determine whether differences in progression of degeneration are indeed a consequence of different temporal patterns of degeneration in the spinal cord compared to the brain, or whether this observation arises from different methodologies. It is tempting to hypothesize that axonal degeneration in hA53Ttg mice starts in the spinal cord and spreads into the brain starting with the brainstem, but further research is needed to confirm this hypothesis. Increased NF-L levels were also reported in 12-months old Line 61 mice overexpressing wild type α-syn under control of the Thy1 promoter (Loeffler et al., 2020). However, the total amount of NF-L in hA53Ttg animals at 10 months of age is more than 3-fold higher compared to Line 61 mice. It therefore appears that the A53T mutation accelerates neurodegeneration in hA53Ttg mice, consistent with findings in patients where the A53T mutation causes early-onset PD (Polymeropoulos et al., 1997; Bozi et al., 2014). We also analyzed tyrosine hydroxylase in the striatum and substantia nigra but could not detect any differences between hA53Ttg animals and age-matched controls (data not shown). This is in accordance with previous reports (Chandra et al., 2005; Rothman et al., 2013). To our knowledge, the only group that reported degeneration of dopaminergic neurons is Martin et al. (2014). However, they reported degeneration in “end-stage” animals at an age of 12 months and thus much older as animals in our study. In summary, our study highlights axonal degeneration as a main pathology in the hA53Ttg model. Additionally, we show that the clinically relevant NF-L biomarker can be used to track the pathological changes caused by α-syn A53T.

## Conclusion

We were able to show that hA53Ttg mice exhibit progressive motor symptoms, increased levels of hα-syn, highly increased levels of α-syn pSer129 starting at very early age, fiber-like structures in the cortex, increased neuroinflammation and axonal degeneration at later ages. Taken together, our findings show that hA53Ttg mice are a valuable tool for Parkinson’s disease research as it accurately models some of the main features of this devastating disease.

## Data Availability

The original contributions presented in the study are included in the article/Supplementary material, further inquiries can be directed to the corresponding author.
