# Supplementary material for: Motor deficits and brain pathology in the Parkinson’s disease mouse model hA53Ttg
Source: Front Neurosci. 2024 Sep 20;18:1462041. doi: 10.3389/fnins.2024.1462041 (PMC11450652; doi:10.3389/fnins.2024.1462041)
Supplement: Supplementary file 2 [file Data_Sheet_1.pdf]

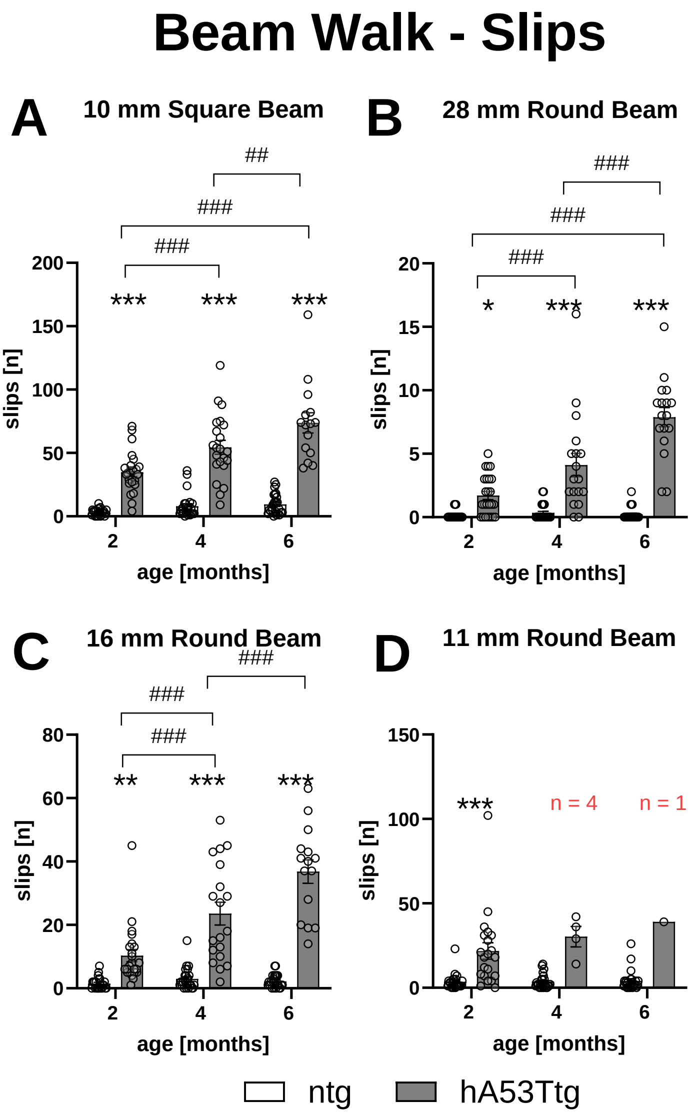

Figure S1: Number of slips on different beams in the beam walk test of hA53Ttg mice. Graphs show a progressive increase in the number of slips of hA53Ttg animals on each of the beams compared to non-transgenic (ntg) controls; *n* = 23-24 per group. Mean + SEM; two-way ANOVA followed by Bonferroni´s *post hoc* test; *p<0.05, **/##p < 0.01, ***/###p < 0.001. *Differences between genotypes; #differences between age groups.

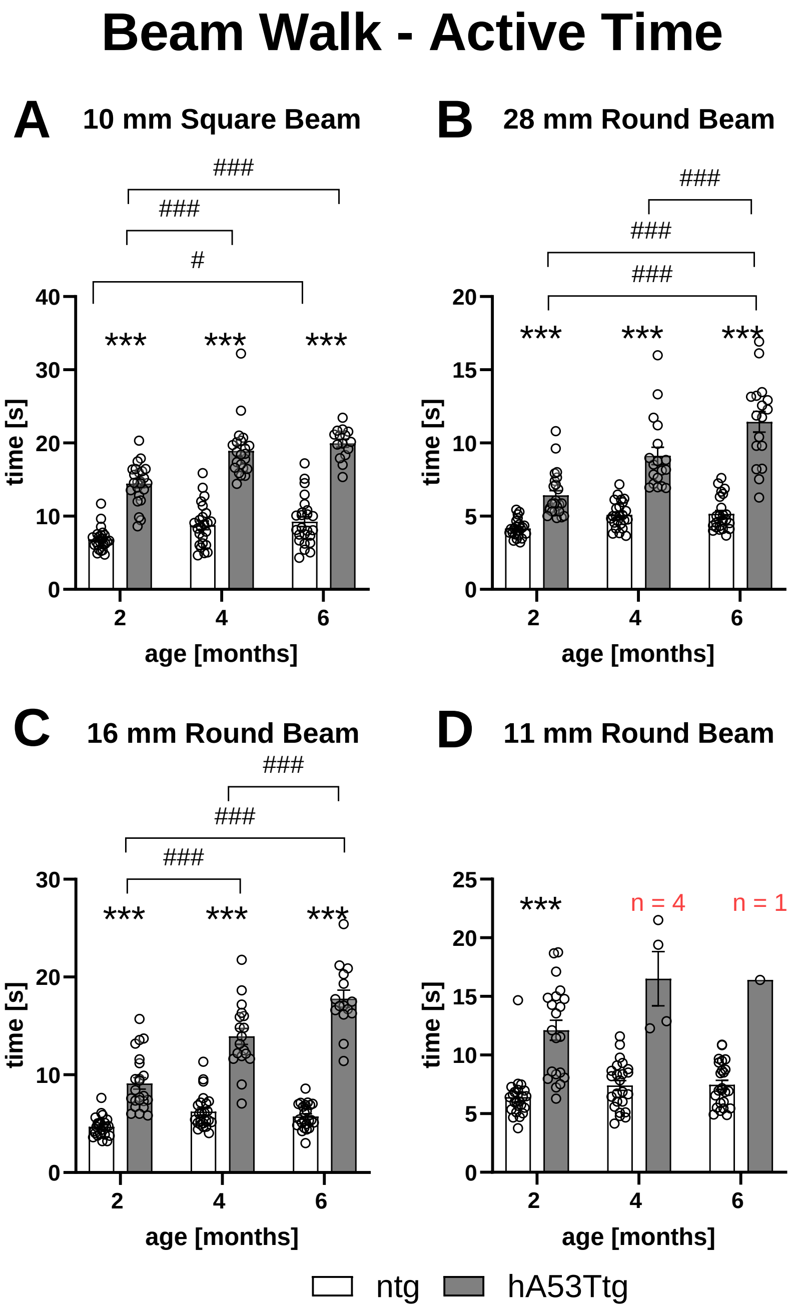

Figure S2: Time needed to cross different beams (active time) in the beam walk test of hA53Ttg mice. Graphs show a progressive increase in the active time of hA53Ttg animals on 4 beams: A: 10 mm square beam, B: 28 mm round beam, C: 16 mm round beam, and D: 11 mm round beam compared to non-transgenic (ntg) controls; n = 23-24 per group. Mean + SEM; two-way ANOVA followed by Bonferroni´s *post hoc* test; #p<0.05, ***/###p < 0.001. *Differences between genotypes; #differences between age groups.

Brainstem - Microgliosis

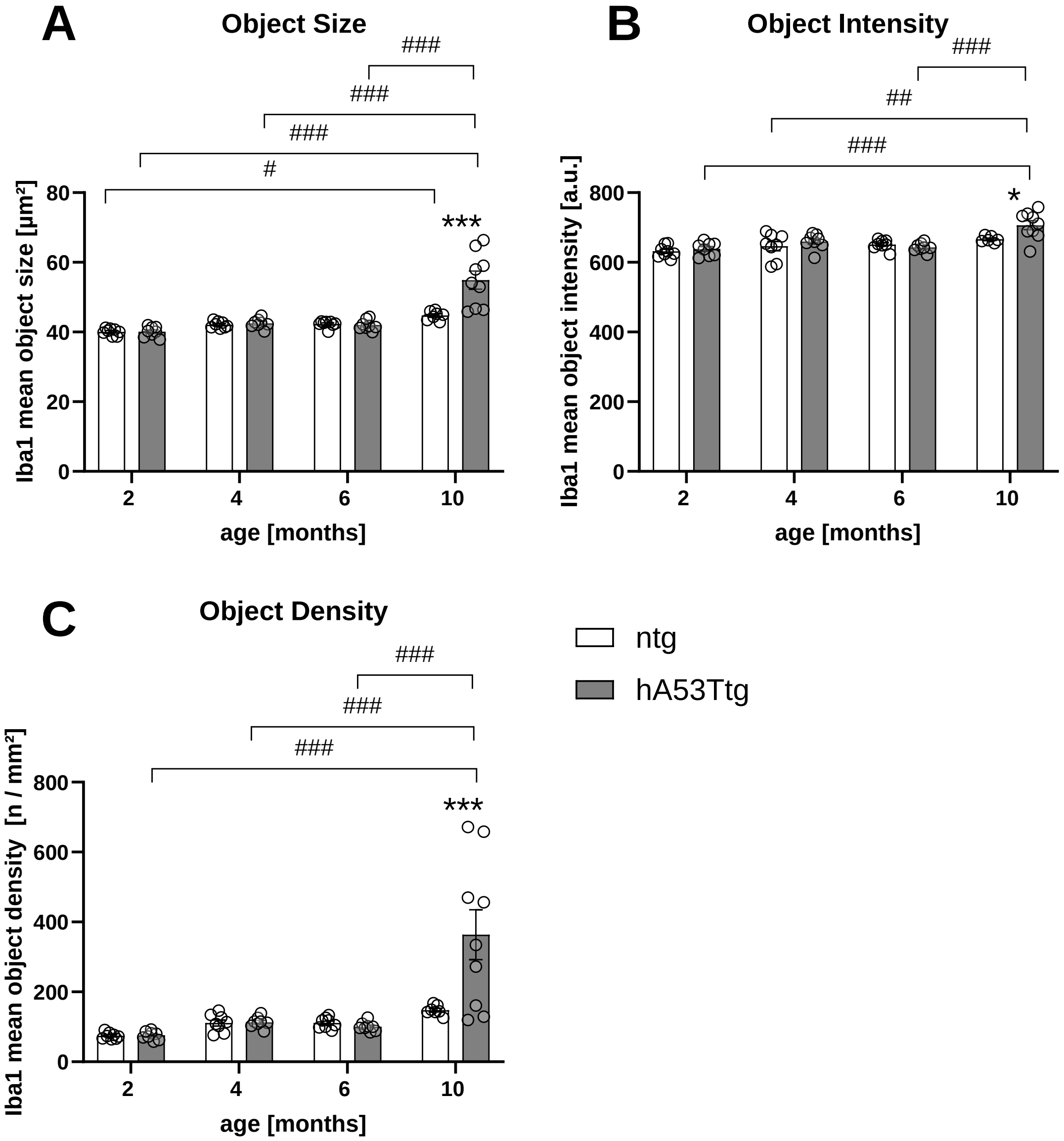

Figure S3: Quantification of Iba1 labeling in the brainstem of hA53Ttg mice. A significant increase in object size (A), object intensity (B) and object density (C) in 10 months old hA53Ttg animals compared to non-transgenic (ntg) controls, as well as compared to 2-, 4-, and 6- month-old animals of the same genotype; *n* = 7-9 per group. Mean + SEM; two-way ANOVA followed by Bonferroni´s *post hoc* test; */#p<0.05, ## p < 0.01, ***/###p < 0.001. *Differences between genotypes; #differences between age groups.

Microgliosis

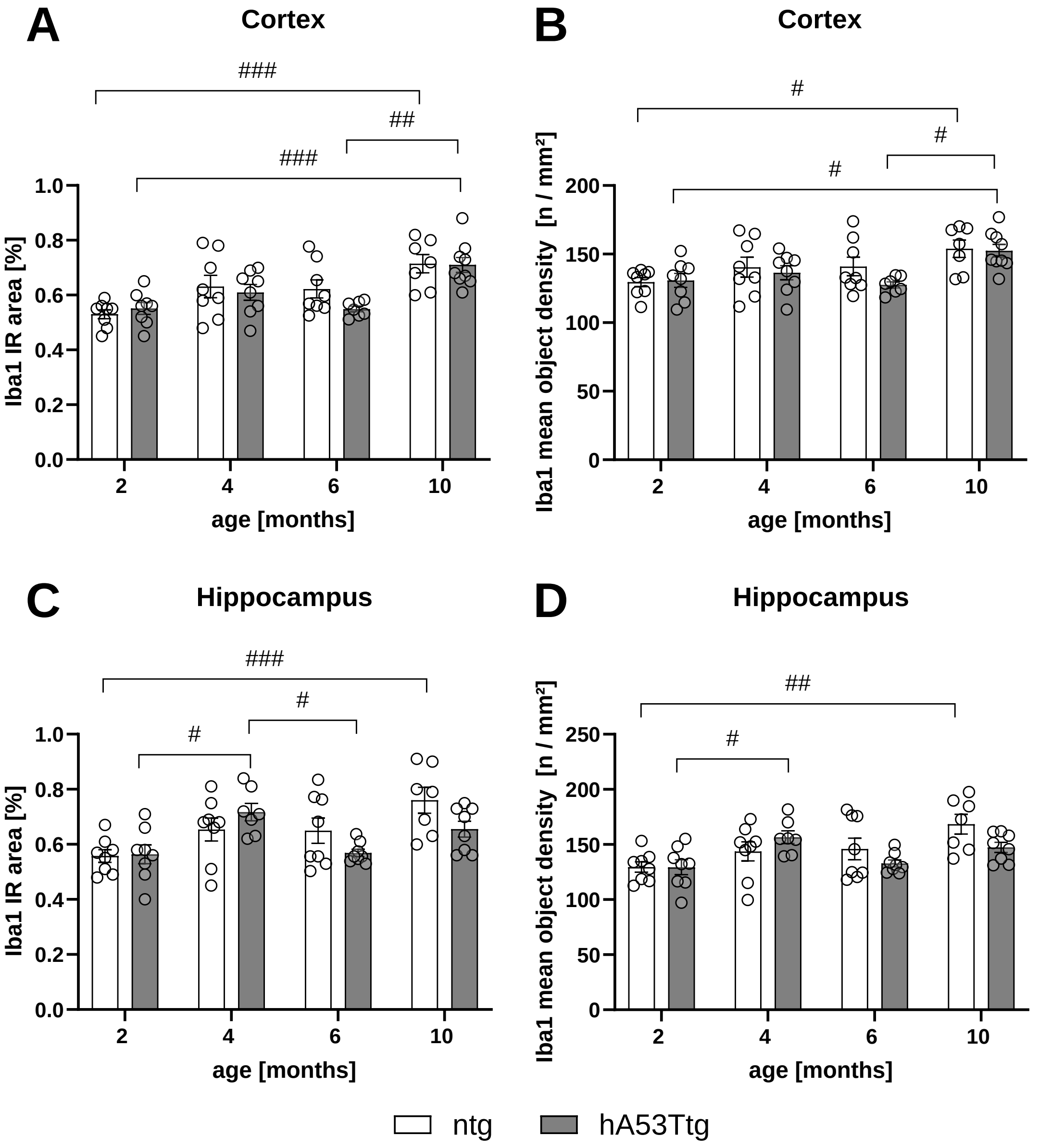

Figure S4: Quantification of Iba1 labeling in the cortex and hippocampus of hA53Ttg mice. Immunoreactive area (A, C) and mean object density (B, D) in the cortex (A, B) and hippocampus (C; D) of hA53Ttg mice. Overall, no effect of the genotype was observed. The differences observed arise from withing group differences; *n* = 7-9 per group. Mean + SEM; two-way ANOVA followed by Bonferroni´s *post hoc* test; #p<0.05, ## p < 0.01, ###p < 0.001. #Differences between age groups.

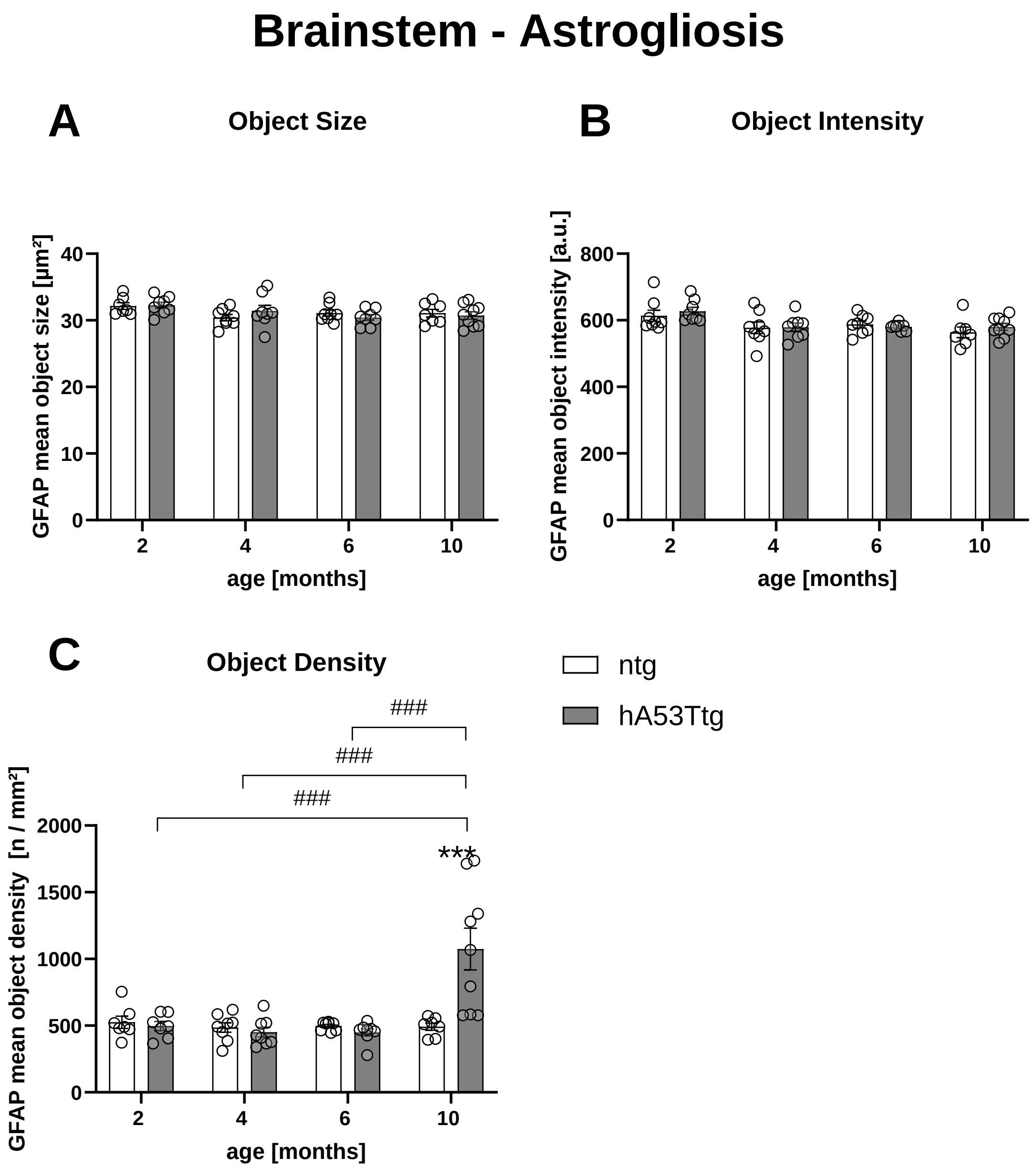

Figure S5: Quantification of GFAP labeling in the brainstem of hA53Ttg mice. No changes in object size (A) or object intensity (B) were observed. An increased object density (C) was observed in 10 months old hA53Ttg animals compared to non-transgenic (ntg) controls, as well as compared to 2-, 4-, and 6- month-old animals of the same genotype; n = 7-9 per group. Mean + SEM; two-way ANOVA followed by Bonferroni´s *post hoc* test; ***/###p < 0.001. *Differences between genotypes; #differences between age groups.

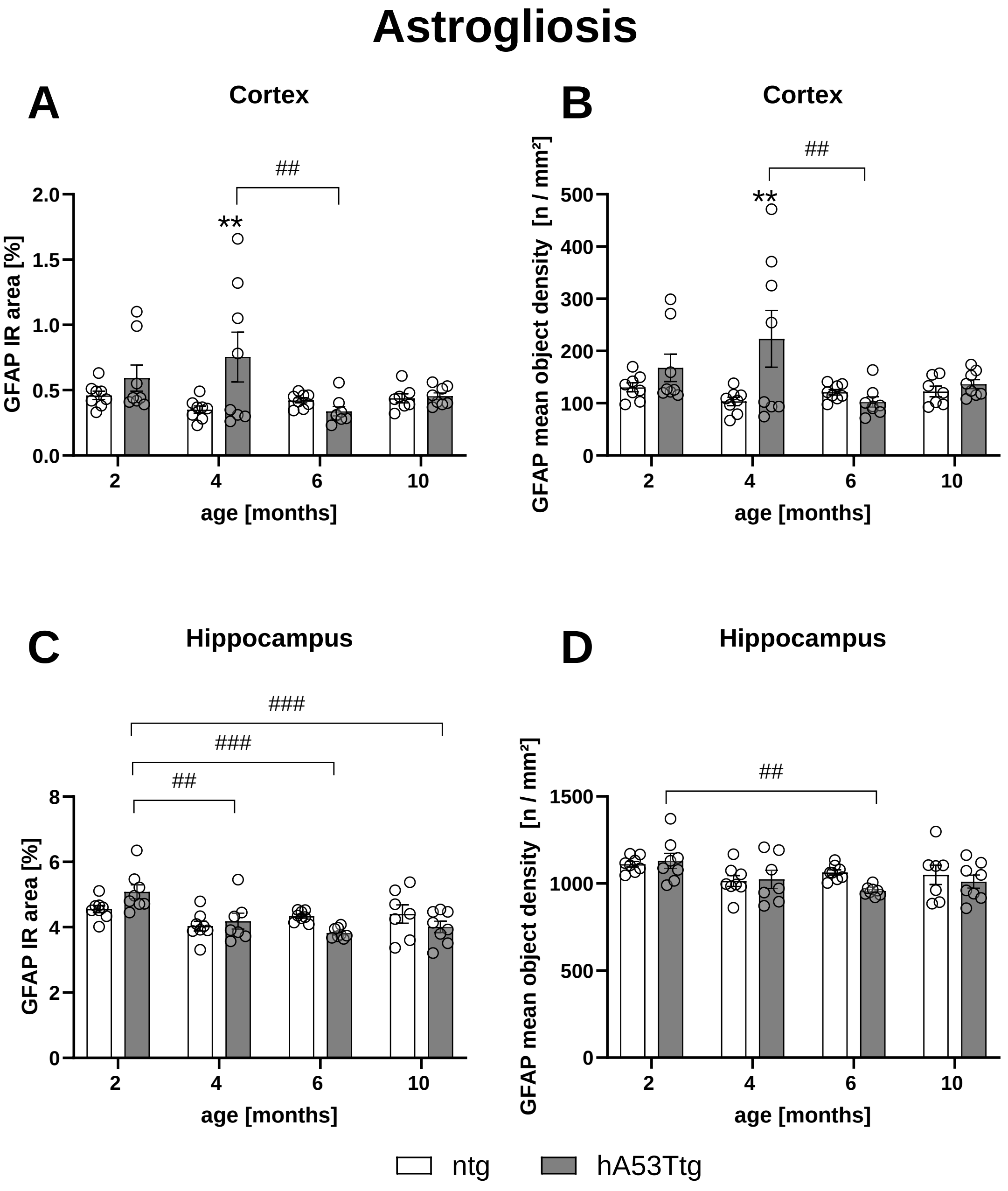

Figure S6: Quantification of GFAP labeling in hippocampus and cortex of hA53Ttg mice. In the cortex, a transient increase in immunoreactive area (A) and object density (B) was observed in 4-month-old hA53Ttg animals compared to non-transgenic (ntg) littermates and compared to 6-month-old transgenic littermates. No significant effect of genotype was observed in the hippocampus (C, D). Differences displayed arise from within group comparisons; n = 7-9 per group. Mean + SEM; two-way ANOVA followed by Bonferroni´s *post hoc* test; **/## $p < 0.01$, ###$p < 0.001$. *Differences between genotypes; #differences between age groups.

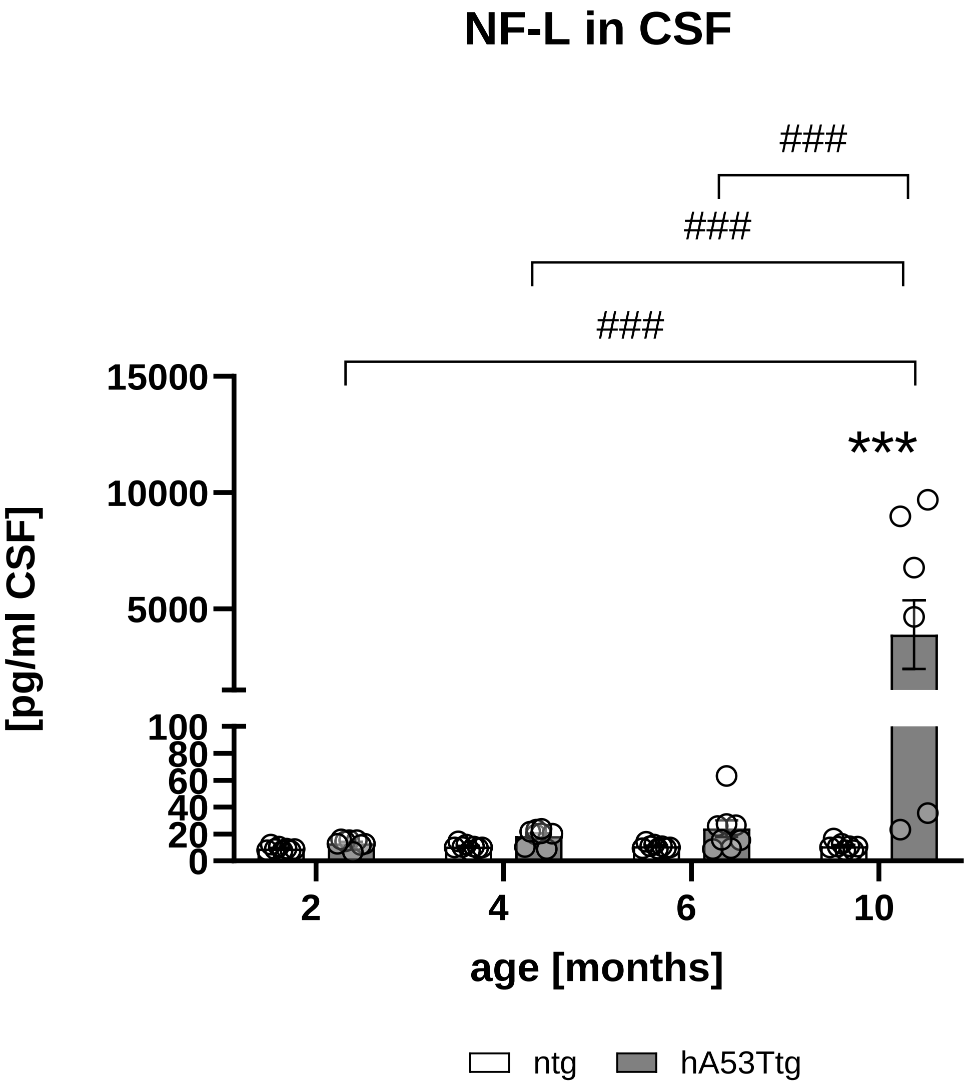

Figure S7: Neurofilament light chain levels in the CSF of hA53Ttg mice. Strongly increased neurofilament light chain (NF-L) levels in the cerebrospinal fluid (CSF) of 10-month-old hA53Ttg animals compared to non-transgenic (ntg) littermates and compared to younger transgenic animals; *n*=8 per group. Mean + SEM; two-way ANOVA followed by Bonferroni´s *post hoc* test; ***/###p < 0.001. *Differences between genotypes; #differences between age groups.
